# Structural heterogeneity and functional convergence of transposable elements

**DOI:** 10.3389/fgene.2025.1605675

**Published:** 2025-12-16

**Authors:** Gleb Yu. Kosovsky, Galina V. Glazko, Tatiana T. Glazko

**Affiliations:** 1 Department of Biotechnology, Afanas’ev Research Institute of Fur-Bearing Animal Breeding and Rabbit Breeding, Moscow, Russia; 2 Department of Biomedical Informatics, University of Arkansas for Medical Sciences, Little Rock, AR, United States

**Keywords:** transposons, regulatory elements, “ancient” and “young” repeats, evolutionarily conserved, spandrels

## Abstract

Almost half the mammalian genomes consist of transposable elements (TEs) and their derivatives. The distribution density of TEs can be associated with genomic regions of chromosomal rearrangements in different mammalian species and with the genomic localization of protein-coding genes that differ in length and function. To evaluate these characteristics at the local genomic level, an analysis of the distribution of various TEs (retrotransposons and DNA transposons) was performed in three mammalian species (human, cattle, and domestic rabbit) in genes with different functions and chromosomal localizations and their flanking regions. In humans and rabbits, melanophilin (MLPH) and myostatin (MSTN) are syntenic, but not in cattle. In the latter, MLPH and the leptin receptor (LEPR) are syntenic, but not in humans and rabbits. The alpha-thalassemia gene is always located on chromosome X. The results indicate that the frequencies of different TEs are species-specific and do not depend on the length of genes, their function, or chromosomal localization. There were also species-specific differences in the ratio of “ancient” and “young” short interspersed nuclear elements (SINEs) and long interspersed nuclear elements (LINEs). There was a statistically significant positive correlation between ancient SINE + LINE and LTR-ERV (p < 0.01) and a significant negative correlation between young SINE + LINE and DNA transposons (p < 0.05). Competitive relationships between TEs are probably defined by the presence of identical regulatory motifs in different TEs, associated with the reliance of TE amplification on the host’s own regulatory systems.

## Introduction

Recent studies have demonstrated that transposon-derived regulatory network elements are associated with phenotypic diversity (Fueyo et al., 2022; Du et al., 2024; Zhao et al., 2023). These studies are particularly important in the era of genome editing, as the structural and functional organization of potential gene editing targets requires careful study to assess possible negative side effects (Kosovsky et al., 2024).

Transposable elements (TEs) contain sequences necessary for their expression and transposition. These sequences can also regulate the host’s transcription, even when the TE itself has lost transposition function because of accumulated mutations. The mechanisms of host transcriptional regulation by TEs include providing enhancer and promoter sequences, targets of three dimensional chromatin organization, transcription factor (TF)-binding sites, and new regulatory elements including non-coding RNAs, the modification of nearby genes’ methylation states, and chromosomal structural modifications (Du et al., 2024). In mammalian genomes, there are gene blocks that are evolutionarily conserved and gene blocks that are frequently subjected to chromosomal rearrangements (homologous synteny blocks (HSBs) and evolutionary breakpoint regions (EBRs)). The differences between HSBs and EBRs are that the latter have high frequencies of TEs and microsatellite loci, more genes with a low number of base pairs, and they are frequently located in regions without topologically associated domains (TAD). HSBs are enriched with genes involved in anatomical morphogenesis and the development of the central nervous system (Damas et al., 2022).

Accumulated data suggest that when searching for genomic elements associated with phenotypic diversity, non-coding sequences with TEs make the greatest contribution, since they can regulate the expression profiles of adjacent genes (Du et al., 2024). TEs of different origin with the same TF-binding sequences are frequently located in syntenic regions. If one of the TEs carrying the corresponding TF-binding nucleotide motif is damaged, then its function can be performed by another TE variant carrying the same motif (Du et al., 2024). The density of TEs carrying TF-binding sites varies significantly between different genomic regions and depends on the localization of genes involved in different pathways (the lowest density is in the regions of genes associated with the early stages of embryonic development) (Damas et al., 2022; Nikitin et al., 2018; Nikitin et al., 2019).

Many studies are focusing on influencing phenotypic characteristics by editing TE elements (e.g., Buttler et al., 2023; Simpson and Chuong, 2023). However, the high evolutionary rate of TEs, as well as their diversity and multifunctionality, make this research avenue difficult.

Mammalian genomes are dominated by TEs and their derivatives that can comprise approximately half of the genome (Farmiloe et al., 2023). They are divided into two groups based on their mobilization intermediates. Class I elements, also known as retrotransposons, mobilize as an RNA intermediate and include autonomous retrotransposons (long interspersed nuclear elements (LINEs)and endogenous retroviruses (ERVs)), as well as non-autonomous short interspersed nuclear elements (SINEs). Class II elements, also known as DNA transposons, mobilize as a DNA intermediate. The diversity and abundance of TEs can be highly different, even for closely related species. One explanation for this pattern is that TEs with lower genomic abundance more strongly influence closely linked host genes and host activity to block TE transcription and insertion at other positions in the genome (Platt et al., 2018).

The difficulties in understanding TE effects on phenotypic diversity are not exclusively related with their extreme diversity and differences in copy numbers; there could also be species-specific TEs influencing the same phenotypic characteristic. For example, exhaustive studies support the hypothesis that different ERVs could be evolutionary driving forces for trophoblast cell fusion and species-specific placental structures (Sakurai et al., 2023). Thus, the same phenotypic characteristic can be formed with convergent participation of different TEs.

In order to elucidate the potential relationship between the function of protein-coding genes and the distribution of various TEs, we here perform a comparative analysis of TEs and the structural and functional features of the genes encoding melanophilin (MLPH, a transporter of melanosomes, in particular), myostatin (MSTN, an intracellular signaling system; a ligand of the transforming growth factor beta), leptin receptor (LEPR, an extracellular signaling system), and alpha-thalassemia (ATRX, involved in cell division mechanisms) in three mammalian species belonging to different orders: *Oryctolagus cuniculus* (domestic rabbit), *Bos taurus* (cattle), and *Homo sapiens* (human). Considering the associations between the functions of protein-coding genes and the distribution of TEs (Damas et al., 2022; Nikitin et al., 2018; Nikitin et al., 2019), one would expect that the lowest TE density would be in the *ATRX* gene, which is always located on chromosome X in mammals.

The analysis of TE distribution in genes and their flanking regions (1 million base pairs long, 1 Mb) includes LINEs, SINEs, endogenous retroviruses (ERVs, long terminal repeats; LTR in what follows), DNA transposons (DNA in what follows) and microsatellites. LINEs/L2 and SINEs/MIRs were considered separately because they are classified as evolutionarily “ancient” repeats compared to LINEs/L1 and SINEs/Alu, tRNA (Smalheiser and Torvik, 2005; Jurka et al., 2007). The subdivision of SINEs and LINEs into “ancient” and “young” transposons is associated with different frequencies of LINEs/L2 and SINEs/MIRs in mammals (monotremes, marsupials, and placentals). Estimates of the monotreme–theria divergence time range between 160 and 210 M years ago; platypus, placed with the echidnas into the Monotremata taxon, has mostly ancient LINEs/L2 and SINEs/MIRs (Warren et al., 2008). The 2.3 Gb platypus genome contains 1.9 and 2.75 million copies of LINE2 and MIR, respectively, compared to significantly lower numbers in other mammals (Warren et al., 2008). It has been shown that ancient LINE/L2s and SINE/MIRs are gradually replaced by young LINE and SINE repeats in mammalian genomes (Buc et al., 2018).

All four genes have distinct functions and belong to gene families of varying sizes and syntenic conservation. Only the *ATRX* gene is consistently located on the X chromosome across mammalian species. Comparative analysis reveals significant differences in the distribution of LINE, SINE, LTR, and DNA transposons across genes and species regardless of the localization and function of the genes considered.

## Materials and methods

For comparative analyses, *O. cuniculus*, GCF_009806435.1, *B. taurus*, GCF_002263795.3, and *H. sapiens*, GCF_000001405.40 (https://www.ncbi.nlm.nih.gov/, GenBank) genome assemblies were used. MLPH, MSTN, LEPR, and ATRX coordinates were uploaded from GenBank (https://www.ncbi.nlm.nih.gov/). *H. sapiens* genome assembly: GRCh38.p14 (GCF_000001405.40). *B. taurus* genome assembly: ARS-UCD1.3 (GCF_002263795.3). *O. cuniculus* genome assembly: mOryCun1.1 (GCF_964237555.1).

The gene neighborhoods were obtained with Gene Advanced Search Builder (https://www.ncbi.nlm.nih.gov/) using the “sort by chromosome” option and “chromosome & organism” query. Evolutionary conservation of genetic linkage analysis for genes in the neighborhoods of MLPH, MSTN, LEPR, and ATRX was performed in genomic segments approximately 2 × 10^6^ bp long, with the reference gene located in the middle of the segment. Non-annotated loci were excluded. The frequencies and distribution of dispersed and microsatellite repeats were found with RepeatMasker software tool (RepeatMasker.org, https://repeatmasker.org/cgi-bin/AnnotationRequest). Genomic TE annotations were obtained from RepeatMasker (https://repeatmasker.org/; *H. sapiens*—RepeatMasker version 406; *B taurus*—RepeatMasker version 405; *O cuniculus*—RepeatMasker version: 405), and correlation coefficients were estimated with https://www.statskingdom.com/correlation-calculator.html.

To estimate the evolutionary conservation of genetic linkage for the reference genes, the distribution of neighborhood genes was analyzed in 2 Mb segments for *H. sapiens, B. taurus*, and *O. cuniculus* genomes. *Neogale vison* (GCF_020171115.1) was also included in this analysis but was excluded from the analysis of dispersed repeats distribution because this species is not represented in the RepeatMasker database.

## Results

To estimate the evolutionary conservation of melanophilin’s genetic linkage, the distribution of neighborhood genes was analyzed in 2 Mb segments for the genomes of *H. sapiens, B. taurus* and *O. cuniculus*. The length (1 Mb) for 5′ and 3′ flanking regions was selected because, on average, the length of each chromatin loop is approximately 1 Mb long. Generally, the length of topologically associated regions varies from 100 kbp to 1 Mb (Cao et al., 2023; Glaser and Mundlos, 2022). We have previously examined the evolutionary conservation of genetic linkage for *MSTN* and *LEPR* genes in various species, including reptiles (Kosovsky et al., 2024).

The results of genetic linkage for MLPH are presented in Table 1. There is consistent genetic linkage between MLPH and *COPS8*, *COL6A3*, *PRLH*, and *RAB17* genes. *PRLH* (enables neuropeptide hormone activity and prolactin-releasing peptide receptor binding activity) and *RAB17* (a key gene regulating melanocytic filopodia formation in melanocytes in mast cells (Beaumont et al., 2011; Babina et al., 2022)) are genetically linked with MLPH even in birds (Bed’hom et al., 2012). All three genes participate in exosome formation and, eventually, in intercellular transport. Two other genes, genetically linked with MLPH, COL6A3 (encodes the alpha 3 chain of type VI collagen, a protein that plays a crucial role in the extracellular matrix), and COPS8 (one of the eight signalosome complex subunits that interact with ubiquitin ligase and is involved in histone modifications) are presumably involved in fundamental processes of intercellular interactions and the differential regulation of gene expression. It is known that E3 ligase triggers extensive gene expression reprogramming by changing global levels of H3K27me3 (a histone modification associated with gene repression) in plants (Wang et al., 2024), bees (Lowe et al., 2022), and in some cellular populations in tumorigenesis (Marine, 2012).

**TABLE 1:** ^a^Comparative analysis of genetic linkage for *MLPH* gene flanking regions in different mammalian species.

Human		American mink		Cattle		Domestic rabbit	
236486410–238555322, Chr 2		43484351–45484351, Chr 3		115959554–118005443, Chr 3		1702392–3739474, Chr 3	
*IQCA*	Motif containing with AAA domain	*AGAP1*	GTP-binding and GTPase-activating protein 43000000–43282943	*IQCA1*	Motif containing with AAA domain	*TWIST2*	Twist family bHLH transcription factor 2
*ACKR3*	Atypical chemokine receptor	*GBX2*	Gastrulation brain homeobox 2	*ACKR3*	Atypical chemokine receptor	*ASB1*	Ankyrin repeat and SOCS box-containing 1
*COPS8-DT*	COPS8 divergent transcript	*ASB18*	Ankyrin repeat and SOCS box-containing 18	*LOC100335382*	Histone–lysine N-methyltransferase SETMAR-like	*TRAF3IP1*	TRAF3-interacting protein 1
*RNU6-1051P*	RNA, U6 Small Nuclear 1051, Pseudogene	*IQCA1*	Motif containing with AAA domain	*COPS8*	COP9 signalosome subunit 8	*PER2*	Period circadian regulator 2
*COPS8*	COP9 signalosome subunit 8	*ACKR3*	Atypical chemokine receptor	*COL6A3*	Collagen type VI alpha 3 chain	*HES6*	Hes family bHLH transcription factor 6
*COL6A3*	Collagen type VI alpha 3 chain	*COPS8*	COP9 signalosome subunit 8	*LOC513039*	40S ribosomal protein S16 pseudogene	*ILKAP*	ILK-associated serine/threonine phosphatase
*MLPH*	237486410–237555322	*COL6A3*	Collagen type VI alpha 3 chain	*MLPH*	116959511–117005449	*ERFE*	Erythroferrone
*PRLH*	Prolactin-releasing hormone	*MLPH*	44484351–44528469	*RAB17*	Member RAS oncogene family	*ESPNL*	Espin
*RAB17*	Member RAS oncogene family	*PRLH*	Prolactin-releasing hormone	*PRLH*	Prolactin-releasing hormone	*SCLY*	Selenocysteine lyase
*RAB17-DT*	RAB17 divergent transcript	*RAB17*	Member RAS oncogene family	*LRRFIP1*	LRR-binding FLII-interacting protein 1	*UBE2F*	Ubiquitin-conjugating enzyme E2F (putative)
*LRRFIP1*	LRR-binding FLII-interacting protein 1	*LRRFIP1*	LRR-binding FLII-interacting protein 1	*RBM44*	RNA-binding motif protein 44	*RAMP1*	Receptor activity-modifying protein 1
*RBM44*	RNA-binding motif protein 44	*RBM44*	RNA-binding motif protein 44	*RAMP1*	Receptor activity-modifying protein 1	*RBM44*	RNA-binding motif protein 44
*RAMP1*	Receptor activity-modifying protein 1	*RAMP1*	Receptor activity-modifying protein 1	*MIR2902*	TNFR1 pathway and apoptosis modulation and signaling	*LRRFIP1*	LRR-binding FLII-interacting protein 1
*LOC124900521*	Small nucleolar RNA SNORD55	*UBE2F*	Ubiquitin-conjugating enzyme E2F (putative)	*UBE2F*	Ubiquitin-conjugating enzyme E2F (putative)	*RAB17*	RAB17%2C member RAS oncogene family
*UBE2F-SCLY*	UBE2F-SCLY readthrough (NMD candidate)	*SCLY*	Selenocysteine lyase	*SCLY*	Selenocysteine lyase	*PRLH*	Prolactin-releasing hormone
*UBE2F*	Ubiquitin-conjugating enzyme E2F (putative)	*ESPNL*	Espin-like	*ESPNL*	Espin-like	*MLPH*	2702392–2739474
*RNU6-1333P*	RNA%2C U6 small nuclear 1333%2C pseudogene	*KLHL30*	Kelch-like family member 30	*KLHL30*	Kelch-like family member 30	*COL6A3*	Collagen type VI alpha 3 chain
*SCLY*	Selenocysteine lyase	*CAMKMT*	Calmodulin-lysine N-methyltransferase	*ERFE*	Erythroferrone	*COPS8*	COP9 signalosome subunit 8
*ESPNL*	Espin-like	*LINC01833*	Long intergenic non-protein-coding RNA 1833	*ILKAP*	ILK-associated serine/threonine phosphatase	*LOC138849152*	YLP motif-containing protein 1-like
*KLHL30*	Kelch-like family member 30	*SIX3-AS1*	SIX3 antisense RNA 1	*HES6*	Hes family bHLH transcription factor 6	*ACKR3*	Atypical chemokine receptor 3
*ERFE*	Erythroferrone	*SIX3*	SIX homeobox 3	*PER2*	Period circadian regulator 2	*IQCA1*	IQ motif containing with AAA domain 1
*ILKAP*	ILK-associated serine	*KRTCAP2P1*	KRTCAP2 pseudogene 1	*TRAF3IP1*	TRAF3-interacting protein 1	*ASB18*	Ankyrin repeat and SOCS box-containing 18
*LINC02610*	Long intergenic non-protein-coding RNA	*LOC124907760*	Keratinocyte-associated protein 2-like	*ASB1*	Ankyrin repeat and SOCS box-containing 1	​	​
*TARDBPP3*	TARDBP pseudogene 3	*SIX2*	SIX homeobox 2	​	​	​	​
*HES6*	Hes family bHLH transcription factor 6	*LINC01121*	Long intergenic non-protein-coding RNA 1121	​	​	​	​
*PER2*	Period circadian regulator 2	*SRBD1*	S1 RNA-binding domain 1	​	​	​	​

^a^ First table raw indicates species; second, coordinates of the analyzed segment (2 Mb in length). Color highlights the four genes with evolutionary conserved genetic linkage for the reference species.

### TEs within *MLPH*, *MSTN*, *LEPR*, and *ATRX* genes

**FIGURE 1:**
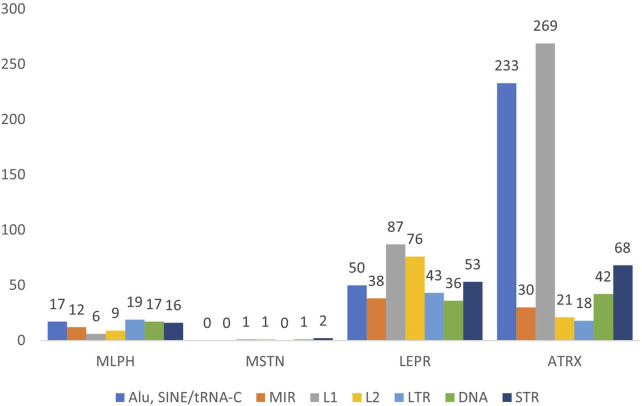
Number of the interspersed (SINE: Alu, MIR; LINE: L1, L2; LTR; DNA) and tandem (STR) repeats in human genes *MLPH, MSTN, LEPR*, and *ATRX*.

**FIGURE 2:**
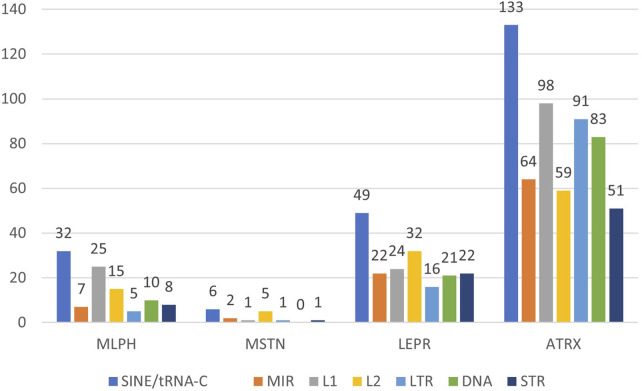
Number of the interspersed (SINE: tRNA-C, MIR; LINE: L1, L2; LTR; DNA) and tandem (STR) repeats in cattle genes *MLPH, MSTN, LEPR*, and *ATRX*.

**FIGURE 3:**
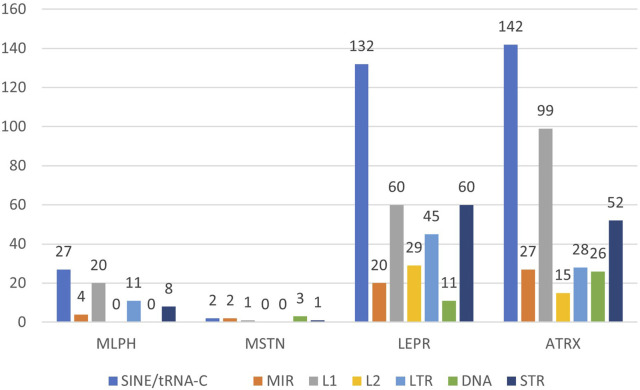
Number of the interspersed (SINE: tRNA-C, MIR; LINE: L1, L2; LTR; DNA) and tandem (STR) repeats in rabbit genes *MLPH, MSTN, LEPR*, and *ATRX*.

Figures 1–3 present comparative analysis of dispersed and microsatellite repeat distributions in *MLPH*, *MSTN*, *LEPR*, and *ATRX* genes in humans, cattle, and rabbits. SINE and LINE repeats are classified as “young” and “ancient”. There are young primate-specific SINE/Alu, and in all species—SINE/tRNA, young LINE/L1, and ancient SINE/MIR and LINE/L2. This classification is subjective because these groups can also be subdivided into relatively recent and relatively ancient repeats, as well as endogenous retrovirus repeats (LTR group) and DNA transposons (DNA group). SINE/Alu repeats are present in primate genomes, while SINE/MIR can be found even in non-mammalian species (Jurka et al., 2007). LINE/L1 is younger than LINE/L2 and actively transposes, while L2 repeats are ancient.

In humans, the length of selected genes is very different, the longest being *ATRX*, then *LEPR*, *MLPH*, and the shortest, *MSTN*. The frequency of repeats in *LEPR* and *ATRX* is also different: approximately twice for LTR, four times for SINE, and almost twice for LINE and microsatellites (STR) (Figure 1). The ratio of ancient and young repeats is approximately the same for three genes; however, for *ATRX*, young LINE L1 is more frequent. *LEPR* (220908 bp) and *ATRX* (281336 bp) have similar lengths; however, the latter has more SINEs and STRs (Figure 1). The shortest gene, *MSTN*, has the lowest frequency of repeats (Figure 1). Interestingly, the frequency of young (SINE/Alu) and ancient (SINE/MIR and LINE1/2) repeats is approximately the same for all genes except *ATRX*, where LINE/L1 was three times more frequent than LINE/L2.

The same comparative analysis of dispersed repeats’ frequency and distribution was performed for cattle (Figure 2). For cattle, gene lengths are much smaller than for humans (except *ATRX*); however, the number of repeats is approximately the same, except that *MSTN* has more repeats than humans. *ATRX* has more MIR, L2, LTR, and DNA in cattle than in humans, despite very similar length (281336 bp in humans and 286037 bp in cattle), unlike *MLPH* (68913 bp in humans and 39463 bp in cattle) and *LEPR* (220908 bp in humans and 104345 bp in cattle).

*MLPH*, *LEPR*, and *ATRX* in cattle, humans, and rabbits also have different exon numbers. *MLPH* has 20 exons in humans, 17 in cattle, and 16 in rabbits; *LEPR* has 20 exons in humans, 21 in cattle, and 27 in rabbits. *ATRX* has 35 exons in humans, 38 in cattle, and 41 in rabbits (GenBank, ncbi.nlm.nih.gov). For all species, *MSTN* had only three exons (Figures 1–3).

In human, *MLPH* and *LEPR* are in different autosomes (Chr 2 and 1), and in cattle they are in the same autosome, Chr 3. In human and rabbit genes, *MLPH* and *MSTN* are in the same autosomes (Chr 2 in human and Chr 3 in rabbit), but in different autosomes in cattle.

*MLPH* and *MSTN* are shorter in rabbits and cattle than in humans, reflected in their exon numbers (*MLPH*: human, cattle, rabbit – 20, 17, and 16, respectively). Despite *ATRX* being shorter in rabbits than in humans and cattle, it has more exons (41 in rabbits vs. 35 in humans and 38 and cattle). *LEPR* (27 exons) and *ATRX* (41 exons) in rabbits have more exons than these genes in humans and cattle (*LEPR*: 20 and 21, *ATRX*: 35 and 38, in humans and cattle, respectively). In addition, in these two genes, rabbits have more SINEs than other species, except *ATRX* in humans (Figures 1–3). This observation is in agreement with the recent finding that SINE TEs are more prevalent in the domestic rabbit genome compared to other livestock species (Zhao et al., 2023). Furthermore, the number of exons in these genes (except MSTN with just three exons) was different for all species. *MLPH* has 20, 17, and 16 exons in humans, cattle, and rabbits; *LEPR* has 20 exons in humans, 21 in cattle, and 27 exons in rabbits; *ATRX* has 35 exons in humans, 38 in cattle, and 41 in rabbits.

The number of different repeats varies from gene to gene, independently of chromosome localization, and depends only on the given gene and species (Table 2). The number of young (SINE/Alu, LINE/L1) and ancient (SINE/MIR, LINE/L2) ТЕs in humans is similar for all genes, except for more LINE/L1 in *ATRX*. In contrast, this gene in cattle has more young repeat SINE/tRNA, while rabbits have more repeat SINE/tRNA not only in *ATRX* but also in *LEPR* (Table 2).

**TABLE 2:** Number of repeats in human, cattle, and rabbit genes (melanophilin (*MLPH*), myostatin (*MSTN*), and *ATRX*). Numbers indicate number of repeats. STR stands for microsatellites (https://repeatmasker.org/cgi-bin/AnnotationRequest).

Species	Gene	SINE		LINE		​	​	​
Gene’s length, bp		Alu, SINE/tRNA-C	MIR	L1	L2	LTR	DNA	STR
Human		Genome/assembly: human - December 2013 - hg38						
68913	*MLPH*	17	12	6	9	19	17	16
7030	*MSTN*	0	0	1	1	0	1	2
220908	*LEPR*	50	38	87	76	43	36	53
281336	*ATRX*	233	30	269	21	18	42	68
Cattle		Genome/assembly: cattle – October 2011 – bostau7						
39463	*MLPH*	32	7	25	15	5	10	8
6627	*MSTN*	6	2	1	5	1	0	1
104345	*LEPR*	49	22	24	32	16	21	22
286037	*ATRX*	133	64	98	59	91	83	51
Rabbit		Genome/assembly: rabbit – April 2009 – OryCun2						
37082	*MLPH*	27	4	20	0	11	0	8
4909	*MSTN*	2	2	1	0	0	3	1
213705	*LEPR*	132	20	60	29	45	11	60
201272	*ATRX*	142	27	99	15	28	26	52

Adelson et al. (2009) found a statistically significant correlation between the ancient SINE/MIR and the ancient LINE/L2 in a whole-genome analysis of cattle. They suggested that this is because non-autonomous SINE/MIR uses the autonomous LINE/L2 for transpositions. Gene- and species-wide differences in their ratio, as well as in the ratio between young SINE and LINE (Table 2), suggest that there are other factors influencing these ratios. These factors could be the presence of motifs that are included in regulatory networks—for example, the motif for transcriptional regulatory factor CTCF (responsible for chromatin higher order structure) (Choudhary et al., 2020; Sharif et al., 2023; Hansen et al., 2024) and G4 quadruplexes associated with regulatory elements (Zhang et al., 2024) that are found in different ТЕs. Recently, it was shown that one TE with CTCF motif can be exchanged for another TE but with the same motif, and the exchange does not disrupt chromatin loop organization (Ivancevic et al., 2018). It was found in the same study that orthologous chromatin loops in mice and humans employ TEs of different structural and evolutionary origin (SINE/B2 in mice, LTR-ERV and DNA endogenous viruses in human), with only motifs for CTCF binding being in common (Choudhary et al., 2020). Currently, there are many data demonstrating that similar regulatory elements are present in different transposons involved in regulatory networks which could influence the association or balance between the numbers of different TE repeats (Du et al., 2024).

In summary, among all genes considered, *MLPH* and *LEPR* are almost twice longer in humans than in cattle, and there are virtually no differences in length for *MLPH* between cattle and rabbits and in *LEPR* between humans and rabbits (Table 2). The frequencies of repeats were independent of gene length and chromosomal localization; however, their distribution and the ratio of young and ancient repeats (SINE and LINE) were species-specific. It might be expected that there are several factors that influence the structure of genes: natural selection in genomes shaping the number of exons, and the presence of different repeats with similar regulatory elements participating in regulatory networks. The distribution of repeats depends more on gene structure than localization on the same chromosome.

### TEs within flanking regions of genes *MLPH*, *MSTN*, and *LEPR*

**FIGURE 4:**
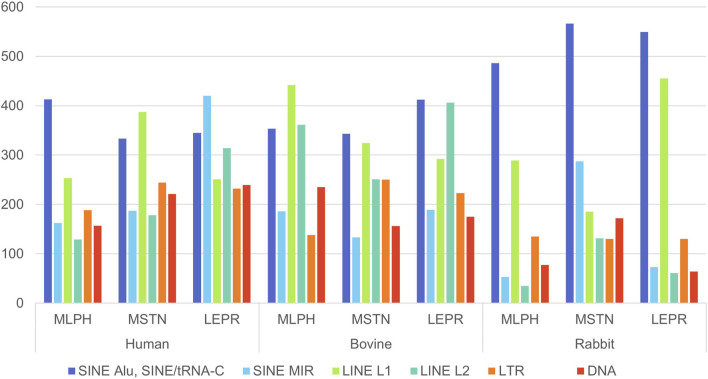
Number of the interspersed (SINE: Alu, tRNA-C, MIR; LINE: L1, L2; LTR; DNA) repeats in 5' (1 Mb) flanking regions of human, cattle, and rabbit genes *MLPH, MSTN* and *LEPR* (Supplementary Materials, Supplementary Table S1, S2, S3).

**FIGURE 5:**
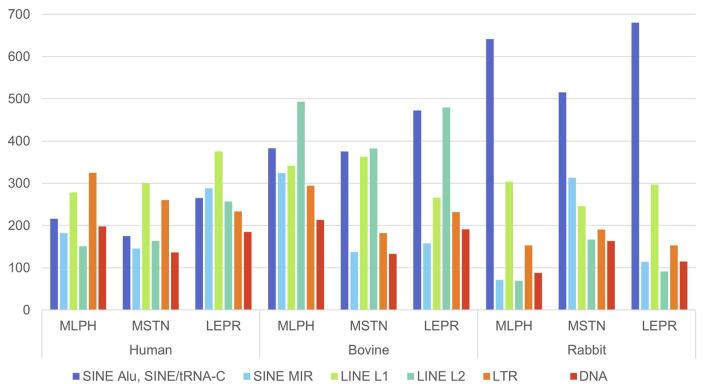
Number of the interspersed (SINE: Alu, tRNA-C, MIR; LINE: L1, L2; LTR; DNA) repeats in 3' (1 Mb) flanking regions of human, cattle, and rabbit genes *MLPH, MSTN* and *LEPR* (Supplementary Materials, Supplementary Table S1, S2, S3).

**FIGURE 6:**
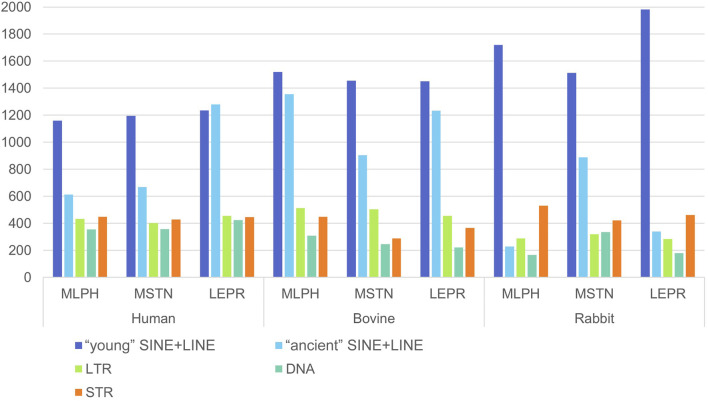
Number of “young” (SINE/Alu and SINE/tRNA + LINE/L1), “ancient” (SINE/MIR + LINE/L2), LTR, DNA transposons and tandem repeats STR in 5'+3' (2 Mb) flanking regions of human, cattle, and rabbit genes *MLPH, MSTN* and *LEPR* (Supplementary Materials, Supplementary Table S4).

The *ATRX* gene was excluded from this analysis because the mammalian X chromosome has unique features, particularly increased enrichment of LINE/L1 (Buc et al., 2018). For each flanking region, a 1 Mb segment length was selected because it is the upper limit of chromosomal loop length, organizing genes in the same structural and regulatory unit (Cao et al., 2023; Glaser and Mundlos, 2022). The results are presented in Figures 4–6.

*MLPH* and *MSTN* human gene flanking regions contain more young SINE and LINE (Figures 4, 5). *LEPR* has more ancient than young repeats in 5′ flanking region (1_mb_1), but in 3′ flanking region (1_mb_2) their frequencies are almost identical. *LEPR* is located in an evolutionarily conserved gene neighborhood and is probably more insulated from repeat insertion than *MSTN*, which does not belong to an evolutionarily conserved gene cluster (Kosovsky et al., 2024). In the flanking regions of all three genes are sequences, homologous to bovine autonomous retrotransposon LINE/RTE-BovB (Supplementary Tables S1–S3) involved in horizontal gene transfer (Ivancevic et al., 2018), and in humans to DNA *Mutator* transposable element that was first described in corn and was later found in mice as an element involved in meiotic recombination (Dupeyron et al., 2019; Underwood and Choi, 2019).

*MLPH* and *MSTN* cattle genes in both flanking regions contain more young than ancient retrotransposons (Figure 6), except for the cattle *MLPH* 2MB_5’+3′ flanking region. Sometimes there were 250–300 more young repeats per 1 Mb than ancient SINE and LINE. For cattle gene *LEPR* flanking regions, the differences between ancient and young repeat frequencies were less drastic than in humans (Figure 6). Unlike the human *LEPR* gene, the cattle gene had more young repeats in flanking regions, but the differences from the ancient were less than for other genes at only approximately 100 repeats (Figure 6). Additionally, SINE/MIR frequencies for cattle were generally less than in humans (Figure 6). That is, there is still the tendency in cattle that young and ancient SINE and LINE frequencies are gene-specific.

For rabbits, repeat frequencies for ancient SINE and LINE were lower than for young repeats. This is probably expected because of the multiple chromosomal rearrangements in the *O. cuniculus* genome (Zhao et al., 2023). Bovine LINE/RTE-BovB repeat frequencies in rabbits were also less than in humans (Supplementary Tables S3).

For some repeats the overall distribution and frequencies were also species-specific (Figure 6). SINE and LINE frequencies were higher for all flanking regions in cattle, and LTR frequencies were higher in *MLPH* and *MSTN* flanking regions than in humans (Figure 6). However, for all flanking regions in cattle, the frequencies of DNA transposons were lower than in humans (Figure 6). Rabbit *LEPR* flanking regions contain more young SINE and LINE repeats than cattle and humans but less LTR and DNA transposons.

The higher frequency of young SINE and LINE repeats in the rabbit than in the cattle genome is in agreement with the results of genomic comparisons in different agricultural species, including rabbits and cattle (Zhao et al., 2023). Lagomorpha and Rodentia belong to the same clade (Glires) and presumably diverged from the last common ancestor approximately 70–65 million years (Fueyo et al., 2022). This allows comparison of the frequencies of young and ancient SINE and LINE repeats in human, cattle, and rabbit genomes with their frequencies in the house mouse genome. The results are presented in Table 3 and suggest that, based on the repeat frequencies, there are two groups of species. In the human and cattle group, the frequencies of ancient SINE/MIR and LINE/L2 repeats (2.9 and 3.7 in human and 2.7 and 2.7 in bovine, respectively) are higher than in the rabbit and mouse group (1.6 and 1.8 in rabbits and 0.6 and 0.4 in mice, respectively). The accumulated data suggest that at the genomic level, the differences in the frequencies of young and ancient DNA repeats and the replacement of ancient repeats with young could be related to differences in litter size (e.g., the size of rabbit and mouse litters is far larger than in humans and cattle), as well as the rate of generational change.

**TABLE 3:** Number of dispersed (non-LTR) and tandem repeats in *Homo sapiens*, *Bos taurus*, *Oryctolagus cuniculus*, and *Mus musculus* genomes (%) (https://www.repeatmasker.org/genomicDatasets/RMGenomicDatasets.html).

Repeats/species	SINE/Alu, tRNA	SINE/MIR	LINE/L1	LINE/L2	STR
*Homo sapiens*	10.5	2.9	17.5	3.7	1.5
*Bos taurus*	9.4	2.7	26.2	2.7	0.9
*Oryctolagus cuniculus*	18.4	1.6	14.9	1.8	1.4
*Mus musculus*	7.2	0.6	19.9	0.4	3.1

The frequencies of ancient and young SINE and LINE repeats in MLPH flanking regions were similar in humans and cattle. This is probably because MLPH genetic linkage with neighborhood genes is more evolutionarily conserved in humans and cattle (Table 1).

There were co-occurrences in all three species of some TEs from different families in *MLPH*, *MSTN*, and *LEPR* flanking regions. To better understand the dependencies between different transposons, we estimated Spearman’s rank correlation coefficients between them.

### Intragroup correlations

Here, for simplicity, we consider conventional *p* < 0.05 as a threshold to indicate “statistical significance”, although we understand it is a controversial and subjective matter (Wasserstein and Lazar, 2016). There were no statistically significant Spearman’s correlations between all ancient and all young repeats, ancient and DNA, young and LTR repeats for *MLPH*, *MSTN*, and *LEPR* flanking regions (*ATRX* was excluded from this analysis because the mammalian X chromosome has unique features that could bias the analysis). However, there were significant correlations between the presence of MIR + L2 and LTR repeats (positive correlation, *r* = 0.862, *p* = 0.003) and L1 and DNA repeats (negative correlation, *r* = −0.767, *p* = 0.026).

Pairwise correlation analysis in nine flanking regions (1 Mb each) for different transposons in three genes resulted in only one statistically significant correlation: between young SINE (SINE/Alu, SINE/tRNA-Core-RTE) and LTR (negative correlation, *r* = −0.883, *p* = 0.004). It should be noted again that the classification into “young” and “ancient” repeats is rather subjective because L1 and MIR groups consist of repeats originating in different times, with different evolutionary histories and species-specific and functional peculiarities.

Next, for the 5′ and 3′ flanking regions (1 Mb length), repeat frequencies were estimated separately (Tables 4, 5). For both the 5′ and 3′ flanking regions, the frequencies of young SINE, ancient SINE, and ancient LINE/L2 were significantly correlated. There were no correlations between the frequencies of young LINE/L1, as well as endogenous retroviruses (LTR). The correlation between DNA transposons for both flanking regions was almost significant (*r* = 0.680, *p* = 0.058).

**TABLE 4:** Distribution of dispersed repeats in 5’ (1 Mb) flanking regions of *MLPH*, *MSTN*, and *LEPR* genes for human, cattle, and rabbit species.

Species	Gene	SINE		LINE		LTR	DNA
Human		Alu, SINE/tRNA-C	MIR	L1	L2		
	*MLPH*	413	162	253	129	188	157
	*MSTN*	333	187	387	178	244	221
	*LEPR*	345	420	251	314	232	239
Cattle	*MLPH*	353	186	442	361	138	235
	*MSTN*	343	133	324	251	250	156
	*LEPR*	412	189	292	406	223	175
Rabbit	*MLPH*	486	53	289	35	135	77
	*MSTN*	566	287	185	131	130	172
	*LEPR*	549	73	455	61	130	64

**TABLE 5:** Distribution of dispersed repeats in 3′ prime (1 Mb) flanking regions of *MLPH*, *MSTN*, and *LEPR* genes for human, cattle, and rabbit species.

Species	Gene	SINE		LINE		LTR	DNA
Human		Alu, SINE/tRNA-C	MIR	L1	L2		
	*MLPH*	216	182	278	151	325	198
	*MSTN*	175	145	300	163	260	136
	*LEPR*	265	288	375	257	233	185
Cattle	*MLPH*	383	324	341	493	294	213
	*MSTN*	375	137	363	382	182	133
	*LEPR*	472	158	266	479	232	191
Rabbit	*MLPH*	641	71	304	69	153	88
	*MSTN*	515	313	246	167	190	163
	*LEPR*	680	114	297	91	153	115

For the 5′ flanking regions (for all genes considered), there were two statistically significant correlations: negative between young SINE and LTR (*r* = −0.930 *p* < 0.001) and positive between ancient LINE/L2 and LTR. In addition, there was almost significant correlation between SINE/MIR and LINE/L2 (*r* = 0.690, *p* = 0.076).

For the 3′ flanking regions, there was almost significant correlation between SINE/MIR and LINE/L2. However, statistically significant correlations were found between SINE/MIR and LTR (*r* = 0.690 *p* = 0.038), SINE/MIR and DNA transposons (*r* = 0.850 *p* = 0.007), and LTR and DNA transposons (*r* = 0.860, *p* = 0.003). Similarly, for 5′ flanking regions, negative correlation between young SINE and LTR (*r* = −0.780 *p* = 0.014) was also found in the 3′ flanking regions.

Despite the limited sample size, structural complexity, and different evolutionary origin of every TE group, the straightforward intragroup correlations that we observed suggest potential cooperative and exclusive associations between the TE groups. Even in the limited genomic regions for different species, we found almost statistically significant positive correlations between SINE/MIR and LINE/L2, presumably because the replication machinery of non-autonomous SINE/MIR ancient retrotransposons depends on the reverse transcriptase of autonomous ancient retrotransposon LINE/L2. Statistically significant negative correlations were also found for two groups: widespread active autonomous retrotransposons LINE/L1 and DNA transposons.

It appears that young SINEs and LINEs, due to their relative activity, disrupt the balance between ancient SINEs, LINEs, and LTR, and DNA TEs and genome sizes.

It should be noted that a certain competition between SINEs, LINEs, and ERVs has been observed in the genomes of South American rodents (Erickson et al., 2011), as well as between SINEs, LINEs, and DNA transposons in bats (Ricci et al., 2023). It can be expected that such interactions may be due to the functional similarity of different TEs in the presence of regulatory motifs and purifying selection against functionally defective TEs, as well as limits to genome expansion.

## Discussion

The *MLPH*, *MSTN*, *LEPR*, and *ATRX* genes of all three species have variable length, number of exons, and dispersed repeat frequencies (non-autonomous retrotransposons SINE, autonomous retrotransposons LINE, endogenous retroviruses LTR-ERV, DNA transposons, and tandem microsatellite repeats, STR) inside the genes and their flanking regions. The number of TEs inside the genes was significantly different: *MSTN* (extensively used for successful gene editing in livestock) had the least amount of TEs and *LEPR* and *ATRX* had the most. There were also significant differences in the ratio of “young” and “ancient” SINE and LINE repeats. For humans and cattle, the balance between SINE and LINE inside *LEPR* gene and in its flanking regions was shifted toward ancient repeats compared to *MLPH* and *MSTN* genes (Figures 1–6; Tables 4, 5).

In this context, it should be noted that only *LEPR* and *ATRX* have gene insertions inside: *LEPR* has the gene *LEPROT* (its sequence overlaps with the LEPR gene itself) and human *ATRX* has *FABP5P15* pseudogene (fatty acid binding protein 5 pseudogene 15, GRCh38.p14, NC_000023.11), as well as long non-coding RNA (lncRNA). In addition, the high frequencies of young SINE and LINE in *LEPR* and *ATRX* suggest they are less insulated from DNA insertions than *MLPH* and *MSTN* (specifically *MSTN*, Figures 1–3). Moreover, *LEPR* belongs to an evolutionarily conserved gene cluster (Kosovsky et al., 2024), and *ATRX* in mammals is always located on the X chromosome. Thus, TEs can also target evolutionarily conserved gene blocks, not only the evolutionarily “fragile” chromosomal regions. However, the relatively high number of ancient (SINE/MIR + LINE/L2) versus young TEs (SINE/Alu and SINE/tRNA + LINE/L1) in the flanking regions of *LEPR*, compared to the *MLPH* and *MSTN* flanking regions in humans and cattle (Figure 6), could be associated with *LEPR* being in a genetically-linked evolutionarily conserved gene cluster.

Questions about the origin, evolutionary mode, and functions of dispersed repeats in the genome are still largely open, despite more than two decades of active research (Jordan et al., 2003). The origin of dispersed repeats is complex; for example, the SINE/Alu repeat originated from the 7SL RNA sequences, a non-coding RNA that is a key component of the signal recognition particle and is essential for protein secretion and translocation into the endoplasmic reticulum. Another example is SINE/tRNA, which originated from tRNA genes; some other repeats are recombination products and overlap, so their origin is untraceable (Sharma et al., 2024). Domestic rabbits have two families of dispersed repeats—OcuSINEA and OcuSINEB—with their central parts originating from LINE and not tRNA, demonstrating that SINE can coopt LINE’s endonucleases (Yang et al., 2021). However, based on the similar frequencies of SINE/Alu in human and SINE/tRNA in cattle and rabbit genomes and the presence of similar regulatory elements in both repeats, it is reasonable to assume that these repeats could have some exchangeable functionalities. For example, SINE/Alu, other SINEs, and some LTR and DNA transposons (e.g., DNA/hATCharlie) contain the same motif for transcriptional regulatory factor CTCF (responsible for chromatin higher order structure). Even without the CTCF binding motif, the clusters of SINE/Alu and SINE/MIR influence local chromatin organization (Gunsalus et al., 2023). In addition, SINE/MIR, LTR, and SINE-VNTR-Alu form a “transposon code”, providing particular patterns of transcription factor binding sites for regulatory networks (Testori et al., 2012; Trizzino et al., 2017). It should be noted that transcription factors that bind heavily depend on the DNA methylation of their binding sites (Rimoldi et al., 2024), and the methylation itself is an epigenetic silencing mechanism of TEs transcription that has both species-specific and common characteristics in vertebrates (Carotti et al., 2023; Ilık et al., 2024). In addition, SINE, LINE, ERV, and DNA transposons are the major sources of regulatory network dynamics, regulating transcription factors, providing promoter, enhancer, and silencer sequences, histone and microsatellite codes, methylation and imprinting, and different non-coding RNAs (Mandal, 2024). For example, in different mammalian species, not only do all TE groups contain transcription factor-binding sites but also many genes encoding these factors contain different TE groups (Du et al., 2024).

In summary, the accumulated evidence suggests that all transposons, even from different classes, despite their structural differences and evolutionary origin, have common characteristics that are related to the mechanisms of their replication, transposition, genomic integration, involvement in 3D genome organization, and interactions with host genes. The interaction could be at the level of neighboring genes and could also be at varying distances. Thus, the common trait of all different TEs is their ability to participate in the host’s regulatory networks by providing a multitude of different regulatory mechanisms to them. Their integration and distribution are defined not by external factors but by instant need inside the genome organization that can be solved by any available source of regulatory element, at any evolutionary moment, thus optimizing survival and successful reproduction. In other words, TE repeat integration is defined not by group membership but by the availability of a TE with necessary regulatory elements. For example, some TEs play a supporting function in the interphase nucleus architecture (Jordan et al., 2003; Falk et al., 2019). This “internal” genomic adaptation was first defined by the architectural term “spandrel” by Gould and Lewontin (1979) and was later adopted by many others (Koonin, 2011). It can be expected that the accumulation of TEs and their turnover is a consequence of the non-adaptive accumulation and exchange of various “junk” genomic elements, functionally convergent in the presence of regulatory motifs (Koonin, 2016).

In the area of finding the most effective markers associated with phenotypic diversity, this approach could shift attention from finding SNPs in causative loci or finding specific genomic elements toward estimating polymorphism for the specific members of regulatory networks.

## Data Availability

Publicly available datasets were analyzed in this study. These data can be found at: https://www.ncbi.nlm.nih.gov/.

## References

[B1] Adelson D. L. Raison J. M. Edgar R. C. (2009). Characterization and distribution of retrotransposons and simple sequence repeats in the bovine genome. Proc. Natl. Acad. Sci. U. S. A. 106 (31), 12855–12860. 10.1073/pnas.0901282106 19625614 PMC2722308

[B2] Babina M. Franke K. Bal G. (2022). How “neuronal” are human skin mast cells? Int. J. Mol. Sci. 23 (18), 10871. 10.3390/ijms231810871 36142795 PMC9505265

[B3] Beaumont K. A. Hamilton N. A. Moores M. T. Brown D. L. Ohbayashi N. Cairncross O. (2011). The recycling endosome protein Rab17 regulates melanocytic filopodia formation and melanosome trafficking. Traffic 12 (5), 627–643. 10.1111/j.1600-0854.2011.01172.x 21291502

[B4] Bed'hom B. Vaez M. Coville J. L. Gourichon D. Chastel O. Follett S. (2012). The lavender plumage colour in Japanese quail is associated with a complex mutation in the region of MLPH that is related to differences in growth, feed consumption and body temperature. BMC Genomics 13, 442. 10.1186/1471-2164-13-442 22937744 PMC3484014

[B5] Buckley R. M. Kortschak R. D. Adelson D. L. (2018). Divergent genome evolution caused by regional variation in DNA gain and loss between human and mouse. PLoS Comput. Biol. 14 (4), e1006091. 10.1371/journal.pcbi.1006091 29677183 PMC5931693

[B6] Buttler C. A. Ramirez D. Dowell R. D. Chuong E. B. (2023). An intronic LINE-1 regulates IFNAR1 expression in human immune cells. Mob. DNA 14 (1), 20. 10.1186/s13100-023-00308-3 38037122 PMC10688052

[B7] Cao Y. Liu S. Cui K. Tang Q. Zhao K. (2023). Hi-TrAC detects active sub-TADs and reveals internal organizations of super-enhancers. Nucleic Acids Res. 51 (12), 6172–6189. 10.1093/nar/gkad378 37177993 PMC10325921

[B8] Carotti E. Carducci F. Barucca M. Canapa A. Biscotti M. A. (2023). Transposable elements: epigenetic silencing mechanisms or modulating tools for vertebrate adaptations? Two sides of the same coin. Int. J. Mol. Sci. 24 (14), 11591. 10.3390/ijms241411591 37511347 PMC10380595

[B9] Choudhary M. N. Friedman R. Z. Wang J. T. Jang H. S. Zhuo X. Wang T. (2020). Co-opted transposons help perpetuate conserved higher-order chromosomal structures. Genome Biol. 21 (1), 16. 10.1186/s13059-019-1916-8 31973766 PMC6979391

[B10] Damas J. Corbo M. Kim J. Turner-Maier J. Farré M. Larkin D. M. (2022). Evolution of the ancestral mammalian karyotype and syntenic regions. Proc. Natl. Acad. Sci. U. S. A. 119 (40), e2209139119. 10.1073/pnas.2209139119 36161960 PMC9550189

[B11] Du A. Y. Chobirko J. D. Zhuo X. Feschotte C. Wang T. (2024). Regulatory transposable elements in the encyclopedia of DNA elements. Nat. Commun. 15 (1), 7594. 10.1038/s41467-024-51921-6 39217141 PMC11366022

[B12] Dupeyron M. Singh K. S. Bass C. Hayward A. (2019). Evolution of *mutator* transposable elements across eukaryotic diversity. Mob. DNA 10, 12. 10.1186/s13100-019-0153-8 30988700 PMC6446971

[B13] Erickson I. K. Cantrell M. A. Scott L. Wichman H. A. (2011). Retrofitting the genome: L1 extinction follows endogenous retroviral expansion in a group of muroid rodents. J. Virol. 85 (23), 12315–12323. 10.1128/JVI.05180-11 21957310 PMC3209412

[B14] Falk M. Feodorova Y. Naumova N. Imakaev M. Lajoie B. R. Leonhardt H. (2019). Heterochromatin drives compartmentalization of inverted and conventional nuclei. Nature 570 (7761), 395–399. 10.1038/s41586-019-1275-3 31168090 PMC7206897

[B15] Farmiloe G. van Bree E. J. Robben S. F. Janssen L. J. M. Mol L. Jacobs F. M. J. (2023). Structural evolution of gene promoters driven by primate-specific KRAB zinc finger proteins. Genome Biol. Evol. 15 (11), evad184. 10.1093/gbe/evad184 37847041 PMC10653712

[B16] Fueyo R. Judd J. Feschotte C. Wysocka J. (2022). Roles of transposable elements in the regulation of mammalian transcription. Nat. Rev. Mol. Cell Biol. 23 (7), 481–497. 10.1038/s41580-022-00457-y 35228718 PMC10470143

[B17] Glaser J. Mundlos S. (2022). 3D or not 3D: shaping the genome during development. Cold Spring Harb. Perspect. Biol. 14 (5), a040188. 10.1101/cshperspect.a040188 34312246 PMC9159266

[B18] Gould S. J. Lewontin R. C. (1979). The spandrels of San Marco and the Panglossian paradigm: a critique of the adaptationist programme. Proc. R. Soc. Lond B Biol. Sci. 205 (1161), 581–598. 10.1098/rspb.1979.0086 42062

[B19] Gunsalus L. M. Keiser M. J. Pollard K. S. (2023). *In silico* discovery of repetitive elements as key sequence determinants of 3D genome folding. Cell Genom 3 (10), 100410. 10.1016/j.xgen.2023.100410 37868032 PMC10589630

[B20] Hansen T. J. Fong S. L. Day J. K. Capra J. A. Hodges E. (2024). Human gene regulatory evolution is driven by the divergence of regulatory element function in both cis and trans. Cell Genom. 4 (4), 100536. 10.1016/j.xgen.2024.100536 38604126 PMC11019363

[B21] Ilık İ. A. Glažar P. Tse K. Brändl B. Meierhofer D. Müller F. J. (2024). Autonomous transposons tune their sequences to ensure somatic suppression. Nature 626 (8001), 1116–1124. 10.1038/s41586-024-07081-0 38355802 PMC10901741

[B22] Ivancevic A. M. Kortschak R. D. Bertozzi T. Adelson D. L. (2018). Horizontal transfer of BovB and L1 retrotransposons in eukaryotes. Genome Biol. 19 (1), 85. 10.1186/s13059-018-1456-7 29983116 PMC6036668

[B23] Jordan I. K. Rogozin I. B. Glazko G. V. Koonin E. V. (2003). Origin of a substantial fraction of human regulatory sequences from transposable elements. Trends Genet. 19 (2), 68–72. 10.1016/s0168-9525(02)00006-9 12547512

[B24] Jurka J. Kapitonov V. V. Kohany O. Jurka M. V. (2007). Repetitive sequences in complex genomes: structure and evolution. Annu. Rev. Genomics Hum. Genet. 8, 241–259. 10.1146/annurev.genom.8.080706.092416 17506661

[B25] Koonin E. V. (2011). The logic of chance: the nature and origin of biological evolution. Upper Saddle River, New Jersey, 07458, USA: ET Press Science.

[B26] Koonin E. V. (2016). Splendor and misery of adaptation, or the importance of neutral null for understanding evolution. BMC Biol. 14 (1), 114. 10.1186/s12915-016-0338-2 28010725 PMC5180405

[B27] Kosovsky G. Y. Skobel O. I. Glazko T. T. (2024). Potential sources of negative effects of gene editing in animals. Sel’skokhozyaistvennaya Biologiya [Agric. Biol.] 59 (6), 1118–1130. 10.15389/agrobiology.2024.6.1118eng

[B28] Lowe R. Wojciechowski M. Ellis N. Hurd P. J. (2022). Chromatin accessibility-based characterisation of brain gene regulatory networks in three distinct honey bee polyphenisms. Nucleic Acids Res. 50 (20), 11550–11562. 10.1093/nar/gkac992 36330958 PMC9723623

[B29] Mandal A. K. (2024). Recent insights into crosstalk between genetic parasites and their host genome. Brief. Funct. Genomics 23 (1), 15–23. 10.1093/bfgp/elac032 36307128 PMC10799329

[B30] Marine J. C. (2012). Spotlight on the role of COP1 in tumorigenesis. Nat. Rev. Cancer 12 (7), 455–464. 10.1038/nrc3271 22673153

[B31] Nikitin D. Penzar D. Garazha A. Sorokin M. Tkachev V. Borisov N. (2018). Profiling of human molecular pathways affected by retrotransposons at the level of regulation by transcription factor proteins. Front. Immunol. 9, 30. 10.3389/fimmu.2018.00030 29441061 PMC5797644

[B32] Nikitin D. Garazha A. Sorokin M. Penzar D. Tkachev V. Markov A. (2019). Retroelement-linked transcription factor binding patterns point to quickly developing molecular pathways in human evolution. Cells 8 (2), 130. 10.3390/cells8020130 30736359 PMC6406739

[B33] Platt R. N. 2nd Vandewege M. W. Ray D. A. (2018). Mammalian transposable elements and their impacts on genome evolution. Chromosome Res. 26 (1-2), 25–43. 10.1007/s10577-017-9570-z 29392473 PMC5857283

[B34] Ricci M. Peona V. Boattini A. Taccioli C. (2023). Comparative analysis of bats and rodents' genomes suggests a relation between non-LTR retrotransposons, cancer incidence, and ageing. Sci. Rep. 13 (1), 9039. 10.1038/s41598-023-36006-6 37270634 PMC10239488

[B35] Rimoldi M. Wang N. Zhang J. Villar D. Odom D. T. Taipale J. (2024). DNA methylation patterns of transcription factor binding regions characterize their functional and evolutionary contexts. Genome Biol. 25 (1), 146. 10.1186/s13059-024-03218-6 38844976 PMC11155190

[B36] Sakurai T. Kusama K. Imakawa K. (2023). Progressive exaptation of endogenous retroviruses in placental evolution in cattle. Biomolecules 13 (12), 1680. 10.3390/biom13121680 38136553 PMC10741562

[B37] Sharif J. Koseki H. Parrish N. F. (2023). Bridging multiple dimensions: roles of transposable elements in higher-order genome regulation. Curr. Opin. Genet. Dev. 80, 102035. 10.1016/j.gde.2023.102035 37028152

[B38] Sharma H. Valentine M. N. Z. Toki N. Sueki H. N. Gustincich S. Takahashi H. (2024). Decryption of sequence, structure, and functional features of SINE repeat elements in SINEUP non-coding RNA-mediated post-transcriptional gene regulation. Nat. Commun. 15 (1), 1400. 10.1038/s41467-024-45517-3 38383605 PMC10881587

[B39] Simpson D. M. Chuong E. B. (2023). Genetic knockout of TE insertions by CRISPR-Cas9. Methods Mol. Biol. 2607, 369–379. 10.1007/978-1-0716-2883-6_17 36449171

[B40] Smalheiser N. R. Torvik V. I. (2005). Mammalian microRNAs derived from genomic repeats. Trends Genet. 21 (6), 322–326. 10.1016/j.tig.2005.04.008 15922829

[B41] Testori A. Caizzi L. Cutrupi S. Friard O. De Bortoli M. Cora' D. (2012). The role of transposable elements in shaping the combinatorial interaction of transcription factors. BMC Genomics 13, 400. 10.1186/1471-2164-13-400 22897927 PMC3478180

[B42] Trizzino M. Park Y. Holsbach-Beltrame M. Aracena K. Mika K. Caliskan M. (2017). Transposable elements are the primary source of novelty in primate gene regulation. Genome Res. 27 (10), 1623–1633. 10.1101/gr.218149.116 28855262 PMC5630026

[B43] Underwood C. J. Choi K. (2019). Heterogeneous transposable elements as silencers, enhancers and targets of meiotic recombination. Chromosoma 128 (3), 279–296. 10.1007/s00412-019-00718-4 31332531

[B44] Wang W. Kim J. Martinez T. S. Huq E. Sung S. (2024). COP1 controls light-dependent chromatin remodeling. Proc. Natl. Acad. Sci. U. S. A. 121 (8), e2312853121. 10.1073/pnas.2312853121 38349881 PMC10895365

[B45] Warren W. C. Hillier L. W. Marshall Graves J. A. Birney E. Ponting C. P. Grützner F. (2008). Genome analysis of the platypus reveals unique signatures of evolution. Nature 453 (7192), 175–183. 10.1038/nature06936 18464734 PMC2803040

[B46] Wasserstein R. L. Lazar N. A. (2016). The ASA statement on *p*-Values: context, process, and purpose. Am. Statistician 70 (2), 129–133. 10.1080/00031305.2016.1154108

[B47] Yang N. Zhao B. Chen Y. D'Alessandro E. Chen C. Ji T. (2021). Distinct retrotransposon evolution profile in the genome of rabbit (*Oryctolagus cuniculus*). Genome Biol. Evol. 13 (8), evab168. 10.1093/gbe/evab168 34270728 PMC8346653

[B48] Zhang R. Wang Y. Wang C. Sun X. Mergny J. L. (2024). G-quadruplexes as pivotal components of cis-regulatory elements in the human genome. BMC Biol. 22 (1), 177. 10.1186/s12915-024-01971-5 39183303 PMC11346177

[B49] Zhao P. Peng C. Fang L. Wang Z. Liu G. E. (2023). Taming transposable elements in livestock and poultry: a review of their roles and applications. Genet. Sel. Evol. 55 (1), 50. 10.1186/s12711-023-00821-2 37479995 PMC10362595

