# Supplementary material for: Structural heterogeneity and functional convergence of transposable elements
Source: Front Genet. 2025 Dec 16;16:1605675. doi: 10.3389/fgene.2025.1605675 (PMC12748216; doi:10.3389/fgene.2025.1605675)
Supplement: Supplementary file 1 [file DataSheet1.docx]

Supplementary Materials

**Supplementary Table 1.** The number of repeats in 5’ prime (1mb_1) and 3’ prime (1mb_2) flanking regions (1 Mb) for human genes (Chr 2, melanophilin, MLPH, Chr 2, myostatin, MSTN, Chr 1, leptin receptor, Chr X, gene ATRX). The overall number of ‘old’ and ‘young’ repeats is presented in the highlighted Table’ cells. Transposons of other species are highlighted in red: LINE/RTE-BovB bovine, DNA/MULE – DNA *Mutator* transposable element first described in plants (Dupeyron, Singh et al. 2019).

| Genes | Repeats/ coordinates | SINE | LINE | LTR | DNA | STR |
| --- | --- | --- | --- | --- | --- | --- |
| MLPH-  1мб_1 | 236487149-  237485819 | 578  Alu413+MIR162 | 433  L1_253+L2_129 | 188 | 157  DNA/hAT-Charlie 86, DNA/TcMar-Tigger 13, **DNA/MULE – 9**  **(блок)** | 235 |
|  | **LINE/RTE-**  **BovB_5 (блок)** | Alu+L1=413+253=666; MIR+L2=162+129=281 | |  |  |  |
| MLPH-  1мб_2 | 237556145-  238555535 | 403  Alu 216+ MIR182 | 476  L1_278+L2_151 | 325 | 198  DNA/hAT-Charlie - 69, DNA/TcMar-  Tigger - 23 | 213 |
|  | **LINE/RTE-**  **BovB_1** | Alu+L1=216+278=494; MIR+L2=182+151=332 | |  |  |  |
| MSTN-  1мб_1 | 189057821-  190054421 | 327  Alu 175+ MIR145 | 490  L1_300+L2_163 | 260 | 136  DNA/hAT-Charlie - 13, DNA/TcMar-  Tigger - 40 | 240 |
|  | **LINE/RTE-**  **BovB_6** | Alu+L1=175+300=475; MIR+L2=145+163=308 | |  |  |  |
| MSTN-  1мб_2 | 190062729-  191062729 | 528  Alu 333+ MIR187 | 608  L1_387+L2_178 | 244 | 221  DNA/hAT-Charlie - 102, DNA/TcMar-  Tigger - 81 | 188 |
|  | **LINE/RTE-**  **BovB_6** | Alu+L1=333+387=720; MIR+L2=187+178=365 | |  |  |  |
| LEPR-  1мб_1 | 64420652-  65420652 | 771  Alu345+MIR420 | 639  L1_251+L2_314 | 232 | 239  DNA/hAT-Charlie - 171, DNA/TcMar-  Tigger - 64 | 211 |
|  | **LINE/RTE-**  **BovB_4** | Alu+L1=345+251=**596**; MIR+L2=420+314=**734** | |  |  |  |
| LEPR-  1мб_2 | 65641559-  66641552 | 558  Alu265+MIR288 | 684  L1_375+L2_257 | 233 | 185  DNA/hAT-Charlie - 85, DNA/TcMar-  Tigger - 41 | 235 |
|  | **LINE/RTE-**  **BovB_4** | Alu+L1=265+375=**640**; MIR+L2=288+257=**545** | |  |  |  |

**Supplementary Table 2.** The number of repeats in 5’ prime (1mb_1) and 3’ prime (1mb_2) flanking regions (1 Mb) for bovine (Chr 3, melanophilin, Chr 2, myostatin, MSTN, Chr 3, LEPR, leptin receptor). The overall number of ‘old’ and ‘young’ SINE and LINE repeats is presented in the highlighted Table’ cells. STR stands for microsatellites.

| Genes | Repeats/ Coordinates | SINE | LINE | LTR | DNA | STR |
| --- | --- | --- | --- | --- | --- | --- |
| MLPH-  1мб_1 | 115959511-  116959511 | 868  SINE/tRNA-Core- RTE_353+MIR_186 | 825  L1_442+L2_177+ LINE/RTE-BovB_184 | 138 | 155  DNA/hAT-  Charlie_91,  DNA/TcMar- Tigger_27 | 235 |
|  |  | SINE/tRNA-Core-RTE+L1=353+442=795; MIR+L2+BovB=186+177+184=547 | |  |  |  |
| MLPH-  1мб_2 | 116998973-  117998973 | 932  SINE/tRNA-Core- RTE_383+MIR_324 | 885  L1_341+L2_272_RTE-  BovB_221 | 294 | 154  DNA/hAT-  Charlie - 90, DNA/TcMar- Tigger - 19 | 213 |
|  |  | SINE/tRNA-Core-RTE+L1=383+341=724; MIR+L2+BovB=324+272+221=817 | |  |  |  |
| MSTN-  1мб_1 | 5278864-  6278864 | 683  SINE/tRNA-Core- RTE_343+MIR_133 | 594  L1_324+L2_89_RTE-  BovB_162 | 220 | 113  DNA/hAT-  Charlie - 69, DNA/TcMar-  Tigger - 13 | 156 |
|  |  | SINE/tRNA-Core-RTE+L1=343+324=667; MIR+L2+BovB=133+89+162=384 | |  |  |  |
| MSTN-  1мб_2 | 6285491-  7285491 | 710  SINE/tRNA-Core- RTE_375+MIR_137 | 770  L1_363+L2_158_RTE-  BovB_224 | 182 | 134  DNA/hAT-  Charlie - 76, DNA/TcMar-  Tigger - 26 | 133 |
|  |  | SINE/tRNA-Core-RTE+L1=375+363=738; MIR+L2+BovB=137+158+224=519 | |  |  |  |
| LEPR-  1мб_1 | 78733479-  79733479 | 834  SINE/tRNA-Core- RTE_421+MIR_189 | 717  L1_292+L2_173_RTE-  BovB_233 | 223 | 108  DNA/hAT-  Charlie - 55,  DNA/TcMar- Tigger - 20 | 175 |
|  |  | SINE/tRNA-Core-RTE+L1=421+292=713; MIR+L2+BovB=189+173+233=595 | |  |  |  |
| LEPR-  1мб_2 | 79837823-  80837823 | 813  SINE/tRNA-Core- RTE_472+MIR_158 | 768  L1_266+L2_182_RTE-  BovB_297 | 232 | 114  DNA/hAT-  Charlie - 49,  DNA/TcMar- Tigger - 28 | 191 |
|  |  | SINE/tRNA-Core-RTE+L1=472+266=738; MIR+L2+BovB=158+182+297=637 | |  |  |  |

**Supplementary Table 3**. The number of repeats in 5’ prime (1mb_1) and 3’ prime (1mb_2) flanking regions (1 Mb) for rabbit (Chr 3, melanophilin, Chr 3, myostatin, MSTN, Chr 7, LEPR, leptin receptor). The overall number of ‘old’ and ‘young’ SINE and LINE repeats is presented in the highlighted Table’ cells. STR stands for microsatellites.

| Genes | Repeats/ Coordinates | SINE | LINE | LTR | DNA | STR |
| --- | --- | --- | --- | --- | --- | --- |
| MLPH- | 1702392- | 539 | 338 | 135 | 77 | 286 |
| 1mb_1 | 2702392 | SINE/tRNA-  C_486+MIR_53 | L1_289+L2_35 |  | DNA/hAT-Charlie 40,  DNA/TcMar-Tigger 18 |  |
|  | **LINE/RTE-BovB_1** | SINE/tRNA-C+L1=486+289=775; MIR+L2=53+35=88 | |  |  |  |
| MLPH- | 2739474- | 713 | 384 | 153 | 88 | 244 |
| 1mb_2 | 3739474 | SINE/tRNA-C_641+  MIR_71 | L1_304+L2_69 |  | DNA/hAT-Charlie - 50,  DNA/TcMar-Tigger - 13 |  |
|  | **LINE/RTE-BovB_0** | SINE/tRNA-C +L1=641+304=945; MIR+L2=71+69=140 | |  |  |  |
| MSTN- | 49301113- | 844 | 330 | 130 | 172 | 234 |
| 1mb_1 | 50301113 | SINE/tRNA-C_566+ MIR_278 | L1_185+L2_131 |  | DNA/hAT-Charlie - 102,  DNA/TcMar-Tigger - 44 |  |
|  | **LINE/RTE-BovB_1** | SINE/tRNA-C +L1=566+185=751; MIR+L2=278+131=409 | |  |  |  |
| MSTN- | 50306022- | 829 | 433 | 190 | 163 | 188 |
| 1mb_2 | 51306022 | SINE/tRNA- C_515+MIR_313 | L1_246+L2_167 |  | DNA/hAT-Charlie - 96,  DNA/TcMar-Tigger - 33 |  |
|  | **LINE/RTE-BovB_1** | SINE/tRNA-C +L1=515+246=761; MIR+L2=313+167=480 | |  |  |  |
| LEPR- | 105975163- | 624 | 535 | 130 | 64 | 211 |
| 1mb_1 | 106975163 | SINE/tRNA- C_549+MIR_73 | L1_455+L2_61 |  | DNA/hAT-Charlie - 36,  DNA/TcMar-Tigger - 10 |  |
|  | **LINE/RTE-BovB_5** | SINE/tRNA-C+L1=549+455=1004; MIR+L2=73+61=134 | |  |  |  |
| LEPR- | 107188868- | 798 | 409 | 153 | 115 | 250 |
| 1mb_2 | 108188868 | SINE/tRNA- C_680+MIR_114 | L1_297+L2_91 |  | DNA/hAT-Charlie - 59,  DNA/TcMar-Tigger - 21 |  |
|  | **LINE/RTE-BovB_0** | SINE/tRNA-C_680+L1_297=680+297=977; MIR_114+L2=114+91=205 | |  |  |  |

**Supplementary Table 4**. Comparative analysis of repeat frequencies for MLPH, MSTN and LEPR genes flanking regions in bovine (highlighted in green), rabbit (highlighted in blue) and human (highlighted in red). STR stands for microsatellites.

| Гены | **SINE + LINE** | **SINE + LINE** | SINE + LINE | **LTR** | **LTR** | LTR | **DNA** | **DNA** | DNA | **STR** | **STR** | STR |
| --- | --- | --- | --- | --- | --- | --- | --- | --- | --- | --- | --- | --- |
| MLPH | **SINE/tRNA-Core- RTE+L1=1519 MIR+L2+BovB=1354** | **SINE/tRNA-Core- RTE+L1=1720 MIR+L2=228** | **Alu+L1=1160; MIR+L2=613** | 513 | 288 | 432 | **309** | 165 | 355 | 448 | 530 | 448 |
| MSTN | **SINE/tRNA-Core- RTE+L1=1455; MIR+L2+BovB=903** | **SINE/tRNA-Core- RTE+L1=1512; MIR+L2=889** | **Alu+L1=1195; MIR+L2=668** | 504 | 320 | 402 | **247** | 335 | 357 | 289 | 422 | 428 |
| LEPR | **SINE/tRNA-Core- RTE+L1=1451; MIR+L2+BovB=1232** | **SINE/tRNA-Core- RTE+L1=1981; MIR+L2=339** | **Alu+L1=1236; MIR+L2=1279** | 455 | 283 | 455 | **222** | 179 | 424 | 366 | 461 | 446 |
